# Seawater pH Predicted for the Year 2100 Affects the Metabolic Response to Feeding in Copepodites of the Arctic Copepod *Calanus glacialis*

**DOI:** 10.1371/journal.pone.0168735

**Published:** 2016-12-19

**Authors:** Peter Thor, Allison Bailey, Claudia Halsband, Ella Guscelli, Elena Gorokhova, Agneta Fransson

**Affiliations:** 1 Norwegian Polar Institute, Fram Centre, Tromsø, Norway; 2 Akvaplan-niva, Fram Centre, Tromsø, Norway; 3 University of Florence, Florence, Italy; 4 Department of Environmental Science and Analytical Chemistry, Stockholm University, Stockholm, Sweden; Stazione Zoologica Anton Dohrn, ITALY

## Abstract

Widespread ocean acidification (OA) is transforming the chemistry of the global ocean, and the Arctic is recognised as a region where the earliest and strongest impacts of OA are expected. In the present study, metabolic effects of OA and its interaction with food availability was investigated in *Calanus glacialis* from the Kongsfjord, West Spitsbergen. We measured metabolic rates and RNA/DNA ratios (an indicator of biosynthesis) concurrently in fed and unfed individuals of copepodite stages CII-CIII and CV subjected to two different pH levels representative of present day and the “business as usual” IPCC scenario (RCP8.5) prediction for the year 2100. The copepods responded more strongly to changes in food level than to decreasing pH, both with respect to metabolic rate and RNA/DNA ratio. However, significant interactions between effects of pH and food level showed that effects of pH and food level act in synergy in copepodites of *C*. *glacialis*. While metabolic rates in copepodites stage CII-CIII increased by 78% as a response to food under present day conditions (high pH), the increase was 195% in CII-CIIIs kept at low pH—a 2.5 times greater increase. This interaction was absent for RNA/DNA, so the increase in metabolic rates were clearly not a reaction to changing biosynthesis at low pH *per se* but rather a reaction to increased metabolic costs per unit of biosynthesis. Interestingly, we did not observe this difference in costs of growth in stage CV. A 2.5 times increase in metabolic costs of growth will leave the copepodites with much less energy for growth. This may infer significant changes to the *C*. *glacialis* population during future OA.

## Introduction

Widespread ocean acidification (OA) is transforming the chemistry of the global ocean, and the Arctic is recognised as the region where the earliest and strongest impacts of OA are expected [1–3]. Sea ice melt has low hydrogen-ion (H^+^) buffering capacity and increasing ice melt makes Arctic waters increasingly susceptible to OA [3]. Additionally, increasing Atlantic water inflow carries large amounts of anthropogenic CO_2_ to the Arctic Ocean [4]. Arctic organisms are therefore the first to face the effects of OA and will continue to experience stronger OA in the future [3]. Contrary to cold adapted eurythermal animals, true Polar species show low energetic costs for maintenance at low temperatures. But such low costs also results in a lower capacity for acid-base regulation [5]. Consequently, true Arctic species may be less capable of countering OA. Moreover, Arctic communities are characterized by simpler food webs both in terms of number of trophic levels and diversity on each trophic level. Effects of environmental change on predator-prey interactions are often buffered by niche sharing at both the predator and the prey level [6]. If any particular species is severely impacted, another will be able to take its place. Such buffering is lessened in simple food webs characterised by a few keystone species and fewer predator-prey interaction points [6].

*Calanus glacialis* constitutes a keystone species in the Arctic Ocean and adjacent seas [7–9]. Along the continental shelf this species dominates in terms of biomass, may exert significant grazing pressure on the microplankton community, and is a very important prey item for many Arctic fish species, baleen whales, and marine birds [10–12]. As a consequence, much attention has been given *C*. *glacialis* and its possible future during Arctic change. Previous studies have shown that while naupliar development and growth may not be significantly affected at OA levels down to pH_T_ 7.47 [13], unchanged development was upheld by increasing physiological buffering at decreasing pH: gene expression was significantly altered in groups of genes coding for such important functions as DNA repair and transcription (Bailey et al. submitted). It seems that pH stress is countered by altered gene expression patterns to maintain unchanged developmental rates. Also, Lewis and colleagues [14] have reported increased naupliar mortality at pH_T_ 7.8 of nauplii caught from under the ice in the high Canadian Arctic. Unfortunately, mortality rates were not measured in the Bailey et al. [13] study. Hatching of eggs also seems somewhat affected. Weydmann et al. [15] found that short-term exposure of mothers (7–9 d) at very low pH (pH_NBS_ 6.9) reduced egg hatching success significantly, whereas incubation at levels more closely mimicking predictions for the year 2100 (pH_NBS_ 7.6) did not show any effects. Our recent studies show significant effects on both ingestion rate and metabolic rate (Thor et al. submitted). We found significantly increasing metabolic rates and decreasing ingestion rates with decreasing pH in copepodite stage IV (CIV) but interestingly not in CV. Hildebrandt et al. [16] also found no effects on ingestion rates in CV at pH_T_ 7.21. Also, long term incubations of females have shown no effects at pH_F_ 7.24 on metabolic rate, gonad maturation rate, or mortality [17]. This suggests that responses to OA may vary among developmental stages in *C*. *glacialis*.

The above-mentioned experimental studies have been conducted using satiating food levels and no studies have considered OA effects at realistic levels of prey availability. Copepods experience vast changes, both temporal and spatial, in prey availability, including starvation, and their metabolism varies accordingly [18–20]. Consequently, any reaction to environmental stress such as OA would be overlaid metabolic variation due to varying ingestion rate, which may outweigh the stress reaction itself. However, OA could interact with energy intake and create more complex responses to OA itself. For instance, such interaction may result from compensatory feeding to meet increased energetic demands or impaired gut absorption under OA stress [21]. In the study presented here, we investigated the metabolic effect of OA and its interaction with food availability in *C*. *glacialis*. We measured metabolic rate and RNA/DNA ratio (an indicator of biosynthesis [22]) concurrently in fed and unfed individuals subjected to two different pH levels representative of present day and the “business as usual” IPCC scenario (RCP8.5) prediction for the year 2100 [23].

## Methods

### Collection of copepods

*Calanus glacialis* were caught by oblique tows of a 200 μm WP2 plankton net with a closed cod end in the Kongsfjord, Svalbard (79.0°N, 11.7°E) during July 2014. No specific authorisation was needed for collecting copepods and no endangered species were involved. On deck, the content of the cod end was diluted in 25 L seawater produced from water collected at 80 m. Another study conducted at the time of sampling showed a salinity of approximately 34.9, temperature of 5.5°C, pH_T_ of 8.17 and *p*CO_2_ of 295 μatm in this water regime [24]. Copepods were then transported to a cold room (5°C) at the nearby Kings Bay Marine Laboratory (Ny-Ålesund, Svalbard). *Calanus glacialis c*opepodites stages II-III, and V (hereafter CII-III, and CV) were selected under the stereoscope using cut off plastic Pasteur pipettes keeping all vessels on ice to avoid high temperatures. Copepodites were identified by number of pleopods and abdominal segments. They were distinguished from *C*. *hyperboreus* and *C*. *finmarchicus* copepodites on the basis of size [9], the presence of red pigmentation in the antennules, a characteristic distinguishing *C*. *glacialis* from *C*. *finmarchicus* [25], and the lack of lateral spikes on the distal prosome segment, a characteristic of *C*. *hyperboreus*.

### Copepod incubations

We conducted one experiment with CII-IIIs and two experiments with CVs. For each experiment, copepods were incubated for 7 days in four different treatments: 1) high pH/no food 2) high pH/high food, 3) low pH/no food, and 4) low pH/high food. Batches of incubation water were prepared from 0.3 μm filtered sea water (*fsw*). Water for the high pH treatments was used unchanged whereas water for the low pH treatments was mixed to the target acidity with small volumes of *fsw* acidified to ca. pH 5.5 by bubbling with CO_2_ (Mapcon CO_2_, Yara Praxair, Tromsø, Norway). For food, the cryptophyte *Rhodomonas baltica* were obtained from cultures at the Marine Research Institute’s Austevoll Research Station (Norway) and cultured in f/2 algal growth medium (Bigelow National for Marine Algae and Microbiota) at exponential growth rates. The growth medium was prepared from 0.3 μm filtered seawater (*fsw*). Cultures were maintained at 18°C and a light:dark cycle of 14h:10h. Algae were added to the batch water for a final concentration of 20 000 cells mL^-1^ (equivalent to 800 μgC L^-1^ considering a cell carbon content of ca. 40 pgC cell^-1^ [26]), a concentration sufficient to ensure maximum and invariant rates of ingestion and metabolism at the far end of the copepod functional response [18]. Any bias from variations in algal concentrations during incubations were therefore avoided. Algal concentrations were measured in the incubation batches by cell counts in a 1 mL flat counting chamber under the microscope at 10x magnification.

Three replicate 620 mL bottles were prepared with water from each batch. For each replicate, 10 individuals were pipetted into the bottle using cut off plastic Pasteur pipettes. All bottles were closed making sure no bubbles were present and placed on a slowly rotating plankton wheel (0.5 rpm) at 5°C in dim light. Every other day approximately 500 mL water was removed from each bottle by reverse filtering. A length of pipe fitted with a 200 μm screen at the bottom was inserted into the bottle. A piece of tubing was inserted 4/5 into this pipe and the water was siphoned off. Newly prepared incubation water was then carefully filled into the bottle through the pipe. Samples for total alkalinity (A_T_) and total dissolved inorganic carbon (C_T_) were taken from the incubation water batches at every water change, fixed with HgCl_2_ (60 μL to 250 mL sample) and stored cold and dark for later analysis.

### Water carbon chemistry determination

Water samples were analyzed for A_T_, C_T_, and salinity at the Institute of Marine Research, Tromsø, Norway following the methods described in Dickson et al. [27]. Briefly, C_T_ was determined using gas extraction of acidified samples followed by coulometric titration and photometric detection using a Versatile Instrument for the Determination of Titration carbonate (VINDTA 3C, Marianda, Germany). A_T_ was determined in the water samples from potentiometric titration with 0.1 N hydrochloric acid using the VINDTA 3C. The average standard deviation for C_T_ and A_T_, determined from replicate sample analyses from one sample, was within ±1 μmol kg^-1^. Routine analyses of Certified Reference Materials (CRM, provided by A. G. Dickson, Scripps Institution of Oceanography, USA) ensured the accuracy of the measurements, which was better than ±1 μmol kg^−1^ and ±2 μmol kg^−1^ for C_T_ and A_T_, respectively. Salinity was measured by a conductivity meter (WTW Cond 330i, Germany) with the precision and accuracy of ±0.05.

C_T_, A_T_, salinity, and temperature were used for each sample as input parameters in the CO2SYS software [28] to calculate total hydrogen-ion scale pH (pH_T_) and partial pressure of CO_2_ using the HSO_4_^-^ dissociation constant from Dickson [29], and the carbonate system dissociation constants (K*1 and K*2) estimated by Mehrbach et al. [30], modified by Dickson and Millero [31].

### Metabolic rate measurements

For estimates of specific metabolic rate (*MR*), oxygen consumption rates (*ṀO*_*2*_) were measured on individual CVs or groups of 3–5 CII-CIIIs. Water was siphoned off the bottles as above and copepods gently poured into Petri dishes kept on ice. While sorting copepods, under the stereoscope, water was continuously replenished from the corresponding incubation bottle. Copepods were pipetted into 1.6 mL vials fitted with fluorescent O_2_ reactive foil discs (PSt3 spots, PreSens, Regensburg, Germany) and vials filled with water from the corresponding incubation bottle. Vials were then sealed with Teflon caps and O_2_ concentrations were measured at 0, 3, and 6 h using an optode O_2_ system (Fibox 3, PreSens, Regensburg, Germany). *ṀO*_*2*_ (nmol O_2_ ind^-1^ d^-1^) was calculated by subtracting the average O_2_ depletion rate measured in five controls from the O_2_ depletion rate in each of the copepod containing vials (nmol O_2_ L^-1^ h^-1^) and multiplying by vial volume (L) and 24 h d^-1^. Prior testing of the optode system at 5°C showed a two-minute 95% reaction time, i.e. the period of time taken before the output reached within 5% of the final O_2_ concentration value (as estimated by exponential regression). Therefore, at every sampling event, O_2_ concentration were read for 3 min, and an average of values read during the last minute was used for calculations. Subsequent to the measurements the copepods were transferred to Petri dishes and photographed under the stereoscope for detailed stage determination and measurement of prosome length. Finally, copepods were pipetted into 1.5 mL centrifuge tubes (Eppendorf, Hamburg, Germany) and 1 mL RNA*later* (Qiagen, Hilden, Germany) was added for preservation and later nucleic acid analysis.

Copepod prosome lengths were measured using ImageJ (U. S. National Institutes of Health) and body carbon weights were calculated using a weight/length relationship of W (μgC) = 4.8L (mm)^3.57^ [32]. *ṀO*_*2*_ (nmol O_2_ ind^-1^ h^-1^) were converted to carbon weight specific *MR* (μgC μgC^-1^ d^-1^) by dividing by body mass (μgC ind^-1^), multiplying by a respiratory coefficient of 0.97 mol C mol O_2_^-1^ [33], multiplying by the mole weight of C (0.012 μg nmol^-1^), and multiplying by 24 h d^-1^.

### Nucleic acid analysis

Gut DNA was quantified in CV’s by staining with PicoGreen (Invitrogen, Carlsbad, CA, USA) according to Gorokhova [34]. Using a sharp needle, a pair of ultrafine forceps and a dissecting microscope, we excised guts of the CV individuals. From each copepod, the gut and the degutted body were transferred into separate 1.5 mL centrifuge tubes containing 50 μl and 300 μl extraction buffer (1% sarcosyl in TE buffer), respectively. For the analysis of the earlier developmental stages, intact individuals were placed in 100 μl extraction buffer. Body RNA and DNA were quantified with RiboGreen (Invitrogen, Carlsbad, CA, USA) according to Gorokhova and Kyle [35], with some modifications, as follows: All samples were homogenized for 30 s and subjected to a repeated (×3) sequence of ultrasound (30 s) and ice bath (1 min). Subsequently, samples were allowed to shake for 2 h at room temperature. Total DNA in the gut samples was determined using 15 μl of sample, 85 μl of TE buffer without dye and 100 μl of TE buffer containing PicoGreen diluted 1:200. All solutions were combined in a solid white Costar 96-well microplate (Corning, Corning, NY, USA), incubated at room temperature for 5 min, and fluorescence was read in a FLUOStar Optima (BMG Labtechnologies, Ortenberg, Germany) at 485 nm excitation and 520 nm emission. Fluorescence of samples designated for RNA/DNA ratio analysis was measured using the same equipment and black solid flat-bottom microplates (Greiner Bio One, Kremsmünster, Austria). Plates were scanned with 0.2 s well measurement time and 10 measurements per well two times firstly after incubation at room temperature for 5 min with 70 μL RiboGreen well^-1^ and secondly after digestion with 5 μl RNAase well^-1^ at 37°C for 30 min. On each analytical occasion, wells containing samples, nucleic acid standards, and negative controls were measured concomitantly. Mean standard curve slope ratio (*m*_DNA_/*m*_RNA_), determined according to Caldarone et al. [36], was 1.58.

### Statistical analysis

Water temperature and *p*CO_2_ were compared among experiments and pH treatments by permutational analysis of variance (PERMANOVA) on similarity matrices assembled using Euclidian distances in Primer 6+ [37] using the design: experiment + pH + experiment x pH.

Separately for each experiment, metabolic rates, RNA/DNA ratios, and gut DNA content were also compared among treatments by PERMANOVA on similarity matrices assembled using Euclidian distances. Two to four samples were measured from each replicate bottle, and we therefore applied a nested design: pH + food level + pH x food level + bottle(pH x food level) with pH and food level as fixed factors and bottle as a random factor nested within pH and food level.

All PERMANOVA tests very followed by PERMDISP tests to verify the assumption of homogeneity of multivariate dispersions.

## Results

Average water chemistry during incubations is shown in Table 1. There were no differences in temperature among experiments or pH treatments (2-factor PERMANOVA, experiments: Pseudo F_3,37_ = 0.88, P = 0.40; treatments: Pseudo-F_1,37_ = 0.074, P = 0.89). pH_T_ was significantly different between pH treatments (2-factor PERMANOVA: Pseudo F_1,19_ = 349, P <0.001), and not significantly different among experiments, except for the low pH in CV experiment 1, which was significantly different from the low pH treatments of all other experiments (2-factor PERMANOVA: pair-wise tests P < 0.05).

**Table 1:** Means ± standard deviations of temperature (*T*), salinity (*S*), total alkalinity (*A*_*T*_), total dissolved inorganic carbon (*C*_*T*_), total hydrogen ion scale pH (pH_T_), and CO_2_ partial pressure (*p*CO_2_) during incubations.

	Treatment	T	S	A_T_	C_T_	pH_T_	*p*CO_2_
		*°C*		*μmol Kg*^*-1*^	*μmol Kg*^*-1*^		*μatm*
CII-III	High pH	4.41±0.23	34.68±0.13	2306±7	2136±6	8.110±0.026	335±22
	Low pH	4.49±0.18	34.67±0.24	2303±11	2265±7	7.726±0.031	881±63
CV exp1	High pH	4.94±0.23	34.77±0.13	2312±8	2152±12	8.082±0.023	361±21
	Low pH	4.94±0.23	34.89±0.19	2312±16	2295±11	7.653±0.055	1060±136
CV exp2	High pH	5.03±0.63	34.70±0.17	2307±10	2138±4	8.103±0.028	341±25
	Low pH	5.18±0.91	34.60±0.24	2305±10	2265±7	7.731±0.029	871±58

**Fig 1:**
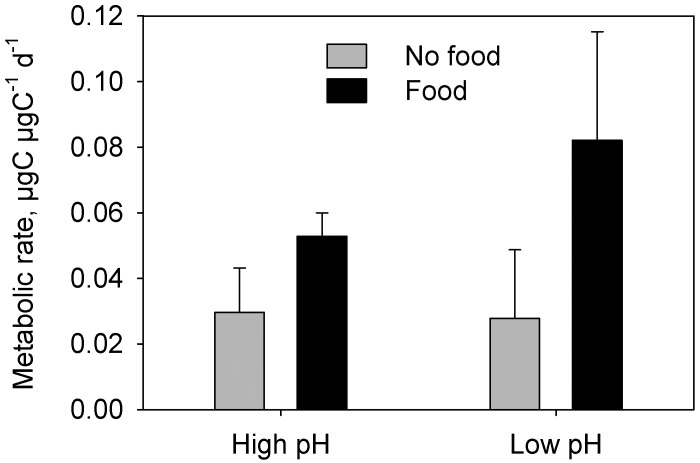
Carbon weight specific metabolic rates (means ± standard deviations) of *Calanus glacialis* copepodite stage II-III.

**Fig 2:**
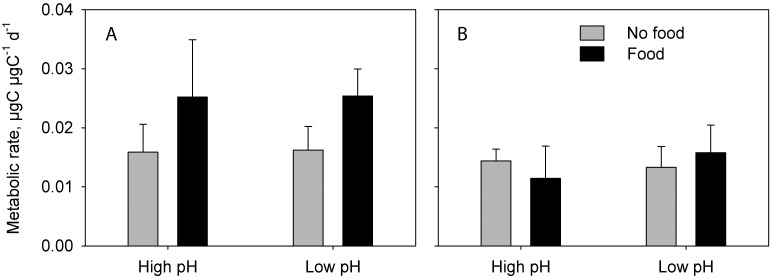
Carbon weight specific metabolic rates (means ± standard deviations) of *Calanus glacialis* copepodite stage V, experiment 1 (A) and experiment 2 (B).

In CII-CIIIs and CV experiment 1, metabolic rate was significantly higher in individuals offered food compared to the un-fed (3-factor PERMANOVA, CII-IIIs: Pseudo F_1,19_ = 24.1, P < 0.001; CVs Exp 1: Pseudo-F_1,8_ = 18.15, P = 0.007; Tables 2 and 3; Figs 1 and 2), whereas CVs in experiment 2 showed no difference in metabolic rate between food levels (3-factor PERMANOVA: Pseudo-F_1,8_ = 0.0013, P = 0.97; Fig 2). pH did not show any main effect on metabolic rate in either CII-CIIIs or CVs, but in CII-IIIs, pH interacted significantly with food level (3-factor PERMANOVA: Pseudo-F_1,19_ = 5.57, P = 0.020; Table 2). While metabolic rates in CII-CIIIs increased by 78% as a response to food in the high pH treatment, the increase was 195% in the low pH treatment (Fig 1).

**Table 2:** *Calanus glacialis* CII-III. Result of the PERMANOVA on metabolic rates.

Source	df	SS	MS	Pseudo-F	P
pH	1	1.48x10^-3^	1.48x10^-3^	2.73	0.115
Food	1	1.32x10^-2^	1.32x10^-2^	24.1	<0.001
pH x Food	1	3.03x10^-3^	3.03x10^-3^	5.57	0.020
Bottle(pH x Food)	19	1.08x10^-2^	5.67x10^-4^	1.66	0.232
Residuals	12	4.10x10^-3^	3.42x10^-4^		
Total	34	3.33x10^-2^			

**Table 3:** *Calanus glacialis* CV experiment 1. Result of the PERMANOVA on metabolic rates.

Source	df	SS	MS	Pseudo-F	P
pH	1	3.51x10^-8^	3.51x10^-8^	0.001	0.967
Food	1	6.30x10^-4^	6.30x10^-4^	18.1	0.007
pH x Food	1	4.91x10^-6^	4.91x10^-6^	0.141	0.731
Bottle(pH x Food)	8	2.79x10^-4^	3.49x10^-5^	1.056	0.422
Residuals	19	6.28x10^-4^	3.30x10^-5^		
Total	30	1.57x10^-3^			

**Fig 3:**
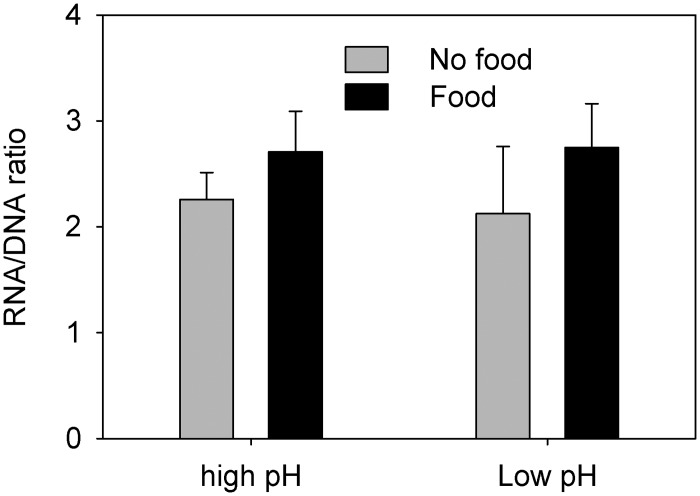
RNA/DNA ratios (means ± standard deviations) in *Calanus glacialis* copepodite stage II-III.

**Fig 4:**
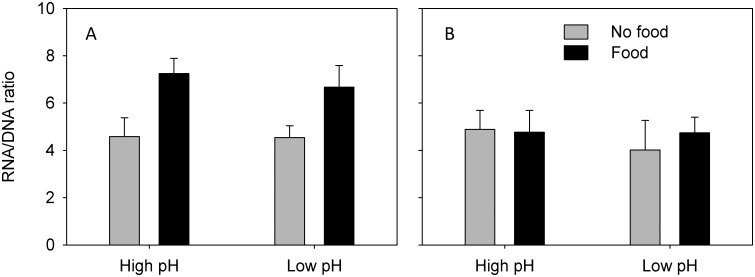
RNA/DNA ratios (means ± standard deviations) in *Calanus glacialis* copepodites stage V, A) experiment 1 and B) experiment 2.

RNA/DNA ratios were significantly different between CII-IIIs offered food and the un-fed (3-factor PERMANOVA: Pseudo F_1,19_ = 12.3, P = 0.004; Fig 3). This was also true for CVs in experiment 1 (3-factor PERMANVOA: Pseudo-F_1,8_ = 63.3, P = 0.001; Fig 4), whereas food level had no effect in CV experiment 2 (3-factor PERMANOVA: Pseudo-F_1,8_ = 0.47, P = 0.5272; Fig 4). There were no significant main effect of pH on RNA/DNA ratios in any of the experiments, and, contrary to metabolic rates, there were no interaction effects of pH and food (3-factor PERMANOVAs: P > 0.05).

**Fig 5:**
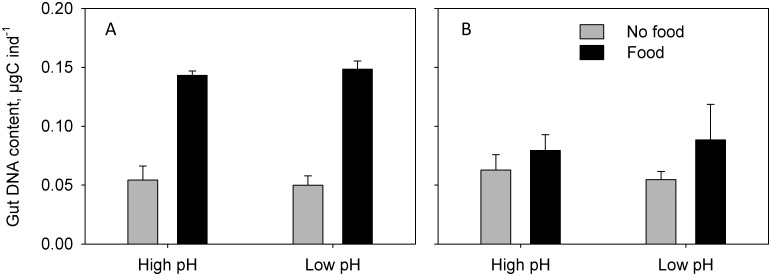
Gut DNA content (means ± standard deviations) in *Calanus glacialis* copepodite stage V, A) experiment 1 and B) experiment 2.

Gut DNA content was significantly different between fed and un-fed CVs in both experiments (3-factor PERMANOVAs: Pseudo-P < 0.05; Fig 5) but there were no effects, main nor interactions, of pH (3-factor PERMANOVAs: Pseudo-P > 0.05). However, in experiment 2 the increase in gut DNA content when offered food was only 27% of that in experiment 1 (Fig 5).

All data are available as S1 Table.

## Discussion

The copepods responded more strongly to the difference in food level than to the difference in pH_T_, both with respect to metabolic rate and RNA/DNA ratio. Copepods show strong functional responses, and significant increases in both metabolic rate and RNA/DNA ratio with prey concentration are not surprising [38–40]. Such observations may provoke the conclusion that future OA effects will be masked by much stronger variations caused by natural temporal and spatial variability of prey concentrations. Our results show, however, that such conclusions are premature, and that effects of pH and food level are not mutually exclusive. We found significant interactions between pH and food level. While metabolic rates in CII-CIIIs were 78% higher in fed than unfed individuals under present day conditions (high pH), the increase was 195% in CII-CIIIs kept at low pH—a 2.5 times greater increase. This interaction was absent for RNA/DNA, so the differences in metabolic rates were clearly not a reaction to changing biosynthesis at low pH *per se* but rather a reaction to increased metabolic costs per unit of biosynthesis. Interestingly, we did not observe this difference in the CVs. In experiment 1, metabolic rates increased by 56% and 58%, in high and low pH respectively, while in experiment 2 we did not observe any increase at all possibly because the copepods fed at very low rates (as indicated by only a minor increase in gut DNA in individuals offered food). Conclusively, the results show that future OA may change the metabolic costs of biosynthesis in *C*. *glacialis* and that this change may be stage dependent.

Previous studies have shown that environmental stress can change copepods’ metabolic costs of biosynthesis. In copepods, the increase in metabolism during feeding (i.e the specific dynamic action; [41]) is caused partly by an increased energetic demand of overcoming fluid drag during the generation of feeding currents [42], but to a larger extent by increased costs of growth associated with protein turnover during digestion, assimilation, and biosynthesis [18, 43]. Accordingly, copepods experiencing prey of poor nutritional quality exhibit significantly lowered specific dynamic action [44]. Maintenance of cellular acid/base balance, the process most likely to be affected by decreasing external pH [45], would normally be assigned a maintenance role, and should as such be unrelated to the costs of growth [46, 47]. Nevertheless, environmental stress may induce significant energetic re-allocation so that resources are moved from anabolic processes involved in growth to accommodate increased maintenance requirements [46, 48] as has been demonstrated in mussels reacting to extreme temperatures [49].

Another calanoid species, *Pseudocalanus acuspes*, have shown interactions between food level and pH similar to the ones found here for *C*. *glacialis* [50]; While a population in the Kongsfjord showed no change due to pH_T_, a population in the boreal Gullmarsfjord, Swedish west coast, showed both main effects of pH and interacting effects with food concentration on ingestion rate and metabolic rate [50]. In this study the magnitude of the specific dynamic action (measured as the slope of the linear relation between rates of ingestion and metabolism) more than doubled in individuals from the boreal population subjected to OA ranging from pH_T_ 7.95 to pH_T_ 7.47. Costs of growth have also been shown to increase during salinity stress. Studies by Calliari et al. [51] have shown significant increases in the ratio between metabolic rate and egg production during salinity stress in the boreal copepod species *Acartia clausi*. OA has been shown to inflict increased protein damage connected to decreased extracellular pH in other crustaceans such as the Norway lobster, *Nephrops norvegicus* [52]. Should such damage also occur in copepods, it could be responsible for increased specific dynamic action because increased damage would lead to increased protein repair, which in turn would incur increased energetic expenses of protein synthesis.

We did not observe any main effects of OA on the metabolism in either developmental stage. Other calanoid copepods have shown significant direct metabolic effects of OA in the range tested here. Metabolic rate increased significantly from pH_NBS_ 8.18 to 7.83 in *Centropages tenuiremis* (no developmental stage indicated) and so did rates in *Pseudocalanus acuspes* females from pH_T_ 8.06 to 7.75 [53, 54]. In *Acartia grani* females, metabolic rates doubled from pH_T_ 8.06 to pH_T_ 7.66 (although low replication rendered the difference non-significant) whereas no clear effect was observed in female *A*. *clausi* exposed to pH_T_ 8.03 and pH_T_ 7.83 in a combined OA and temperature experiment [55, 56]. The lack of response of *C*. *glacialis* CVs in our study have been shown to last during longer term incubations (62 d) of *C*. *glacialis* CVs and *C*. *hyperboreus* CVs and females [17]. Similarly, CVs of the sibling species *C*. *finmarchicus* do not respond to OA in the range tested here. While metabolic rates were found to increase linearly in a very wide range of OA from pH_T_ 8.02 to pH_T_ 7.16 in culture reared CVs of this species [57], this change would not be sufficient to create any significant difference by the OA scenario tested in the present study (pH_T_ 8.10–7.70). Accordingly, wild caught *C*. *finmarchicus* CVs did not show any difference between pH_T_ 7.92 and pH_T_ 7.51 in a later study [58]. To our knowledge, no previous studies have investigated metabolic OA effects in *Calanus* CII-CIIIs.

While the increase in metabolism during feeding was affected by OA in the CII-CIIIs, we did not observe this in the CVs. *Calanus* exhibit many stage specific differences in their metabolism and in this respect the CV stage stands out. While somatic growth is the main purpose for early copepodite stages, metabolism is reconfigured to target lipid storage in CVs for the preparation for hibernation. Hence, the lipid mass of CVs approaching hibernation is ten-fold higher than of CIVs [59]. During hibernation, *C*. *glacialis* CVs may experience extracellular pH approaching 5.5, a possible result of metabolic depression [60]. It is therefore quite conceivable that mechanisms to handle low pH could be activated in this particular stage as part of the general physiological re-organisation to accommodate hibernation. This would render CVs particularly unresponsive to ambient pH. Furthermore, it would be evolutionarily beneficial to avoid the activation of such, possibly costly, mechanisms in earlier developmental stages where they would not be needed.

Even when restricted to specific developmental stages, increased costs of growth may induce reduced life time fitness and thus influence population development [46]. As a rough estimate ca. 70% of assimilated energy is allocated to growth in copepods (0.70 net growth efficiency) [18], with the remainder (30%) used to cover costs of growth by respiration. When *C*. *glacialis* CII-CIIIs experience a 2.5 times higher increase in metabolic costs of growth during OA, 75% (2.5 x 30%) of assimilated energy is lost by respiration with only 25% left for growth. Such diminished growth rates in the early copepodite stages will obviously have significant effects on the development of any copepod population. Certainly, changes in spring production can influence summer biomass. Long-term sampling series in the North Sea have shown that years with low juvenile growth during spring results in lower summer biomass than years with higher juvenile growth [61]. Similar variations have also been observed in the sub-Arctic Pacific *Neocalanus plumchrus* population. This population experiences significant inter-decadal variations in summer biomass [62]. While both differences in the timing of spawning and differences in larval mortality was hypothesised as the origin of these variations, a third hypothesis put forward was that variations in copepodite growth rate influenced the timing of peak biomass among years.

The study presented here accentuate the elusiveness of biological OA effects. Only a few studies have investigated the possibility of interactions of OA effects with food level, but if the relationship found in the present study is common to many species, it makes predictions of general OA responses exceedingly difficult. Moreover, our study shows that OA effects vary not only among taxa, but also among different developmental stages (See also the work by Dupont et al. [63]). This warrant a warning against premature conclusions that specific species are tolerant to OA based on single measurements of specific processes such as fecundity of adults or effects on specific developmental stages without considering effects in different environments (such as e.g. food level) and through the entire ontogenesis.

## Supporting Information

S1 Table Raw data from all three experiments. Metabolic rates (μgC μgC^-1^ d^-1^) and RNA/DNA ratios of CII-IIIs and CVs and gut DNA content (μgDNA ind^-1^) of CVs at the 4 treatments high pH/No food, high pH/Food, low pH/No food, and low pH/Food.
(XLSX)
